# Supplementary material for: Identification of genetic loci for early maturity in spring bread wheat using the association analysis and gene dissection
Source: PeerJ. 2023 Oct 9;11:e16109. doi: 10.7717/peerj.16109 (PMC10569184; doi:10.7717/peerj.16109)
Supplement: Supplemental Information 1 [file peerj-11-16109-s001.pdf]

Table S1 Primer sequences, annealing temperatures, and expected PCR product sizes of candidate genes from locus at 7B. The primers were designed in this study to analyze the expression of candidate genes for maturity time in spring wheat panel.

Gene ID	Primer name	Primer sequence (5’→3’)	Tm (°C)	Amplicon length (bp)
TraesCS7B02G391800	7B_391800_F	CGCAGCTAACTGACCTTGAG	62	120
	7B_391800_R	GGATTCTGTTGTCCGTGACT	62	
TraesCS7B02G396600	7B_396600_F	CCGGAGGTTTGCCTATAGAATTA	62	101
	7B_396600_R	GTCTCCAAGTGAGATCGGAATC	62	
TraesCS7B02G411800	7B_411800_F	GAACATTGAGACAGGCAAGAAC	62	119
	7B_411800_R	CACCACCGCAAGACAATCTA	62	
TraesCS7B02G401600	7B_401600_F	AGAAACATGGGTGGGCTATC	62	82
	7B_401600_R	AAGGCCTTATGGCCCTTATC	62	
TraesCS7B02G400600	7B_400600_F	GCGTCGGGAGCTTAGAGAT	63	139
	7B_400600_R	TCCGGCACCGTCAAGTAT	63	
TraesCS7B02G412200	7B_412200_F	CTGCCACTGAGGTTGAGCTT	64	123
	7B_412200_R	CCACCCATCTGAAGGAGGTTT	64	
TraesCS7B02G399700	7B_399700_F	CTTGCCAGGGAGGTGTATAG	61	135
	7B_399700_R	ATCACCTCGTCCTCTGCC	63	
TraesCS7B02G404300	7B_404300_F	GTCGTCTTCATCCGTTCAGAG	62	78
	7B_404300_R	TTTGGCCGTTGTGTATCCAG	63	
TraesCS7B02G395600	7B_395600_F	TGTTCGCGTATGTCCGATAG	62	109
	7B_395600_R	TTTCCTAGTTGTGCTAAGTAATGTG	61	
TraesCS7B02G400500	7B_400500_F	TCTGCACAGCCGTAAGTA	60	135
	7B_400500_R	GAGAAATCATTGTGATGGAAATC	58	
TraesCS7B02G398700	7B_398700_F	AGACCTGAGCACCGTCTT	63	91
	7B_398700_R	TAGAAGCGCAGATGACTCCA	63	
TraesCS7B02G413400	7B_413400_F	GGGAGTAGTGCTCAACCTG	61	135
	7B_413400_R	CGGTAGTGGTCCTCCGTAT	62	

Table S2 Primer sequences and expected PCR product sizes of reference genes to be tested used in the experiment aimed to analyzing expression of candidate genes for maturity time in spring wheat panel. The primer sequences are from articles dedicated to selecting reference genes with stable expression in wheat (see column “Reference”).

Gene name		Forward Primer (5' → 3')	Reverse Primer(5' → 3')	Amplicon	Reference
Elongation factor 1-alpha	ELF1a_F	CAGATTGGCAACGGCTACG	CGGACAGCAAAACGACCAAG	227	Crismani et al., 2006
60S Ribosomal protein L18a	60S_Rib	CAAGGAGTACCGTGACAC	GCGGGAACTTGATCTTCG	179	Paolacci et al., 2009
Ta.14126.1.S1_at	SAR	GAGTCTGCCCACCCATTCGTAA	GACATGCCATAGGTTTCAGCGAC	155	Long et al., 2010
Ta.7894.3.A1_at	MetAP1	AGCAAGTTGTGACCCGAGGA	GGCGTCAGCAAATAGCAAGTG	115	Long et al., 2010
Ta2291	TaADP-RF	GCTCTCCAACAACATTGCCAAC	GCTTCTGCCTGTCACATACGC	165	Paolacci et al., 2009
Ta2776	RNaseL_ing	CGATTCAGAGCAGCGTATTGTTG	AGTTGGTCGGGTCTCTTCTAAATG	242	Paolacci et al., 2009
Ta54227	CDCP	CAAATACGCCATCAGGGAGAACATC	CGCTGCCGAAACCACGAGAC	227	Paolacci et al., 2009

Table S3 Summary statistics for the maturity time detected during the phenotyping of 92 spring wheat varieties cultivated in the two experimental fields of ICG SB RAS in the Novosibirsk Region from 2005 to 2014 and 2018, spanning eleven years.

	year 2005	year 2006	year 2007	year 2008	year 2009	year 2010	year 2011	year 2012	year 2013	year 2014	BLUEs	2018 Field 1	2018 Field 2
Min	64	66	65	64	70	60	68	63	65	63	67.9	76	74
max	86	88	90	89	91	91	96	81	91	85	83	105	111
range	22	22	25	25	21	31	28	18	26	22	15.1	29	37
sum	6392	6606	6838	6360	7433	7178	7151	6290	6785	6378	6894.2	7780	8137
median	72	76	78	71.5	86	83	81	71	77	72	76.8	90	89
mean	72.64	75.07	77.7	72.27	84.47	81.57	81.26	71.48	77.1	72.48	76.6	90.74	88.45
SE.mean	0.42	0.52	0.45	0.5	0.48	0.51	0.49	0.36	0.41	0.42	0.32	0.76	0.78
CI.mean	0.84	1.04	0.89	1	0.95	1.02	0.97	0.71	0.82	0.84	0.64	1.52	1.56
var	15.64	24.16	17.59	22.41	20.07	23.28	21.02	11.29	14.85	15.63	9.46	50.01	57.13
std.dev	3.95	4.91	4.19	4.73	4.48	4.83	4.59	3.36	3.85	3.95	3.08	7.08	7.56
coef.var	0.05	0.07	0.05	0.07	0.05	0.06	0.06	0.05	0.05	0.05	0.04	0.078	0.085

SE.mean - standard error on the mean, CI.mean - confidence interval of the mean, var – variance, std.dev - standard deviation, coef.var - variation coefficient

Table S4 Analysis of variance (ANOVA) on the data for the phenotyping of the maturity time of 92 spring wheat varieties cultivated in the two experimental fields of ICG SB RAS in the Novosibirsk Region from 2005 to 2014 and 2018.

	Df	Sum Sq	Mean	Sq F	value Pr(>F)
Cultivar	89	8417	94.6	9.568	<2e-16 ***
Year	9	17161	1906.7	192.893	<2e-16 ***
Residuals	801	7918	9.9		

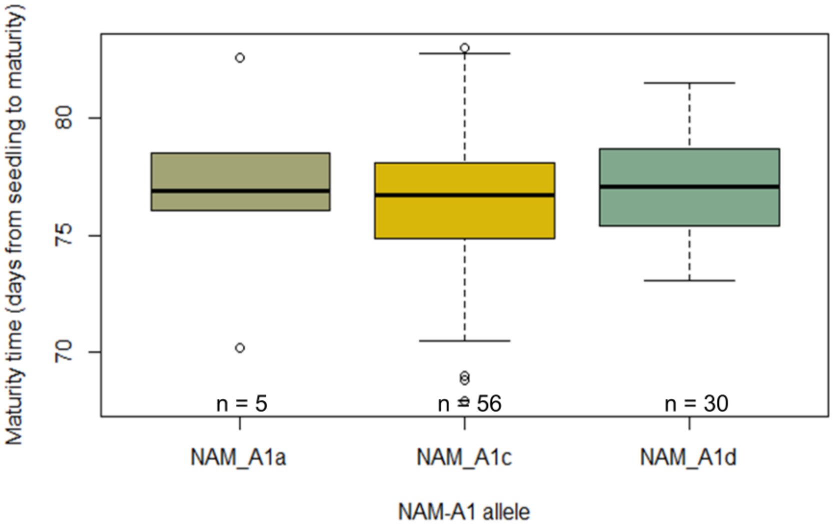

Fig. S1 Boxplots of the maturity time variation for genotypes showing different alleles of *NAM-A1* gene. “n” denotes the number of samples in each group.

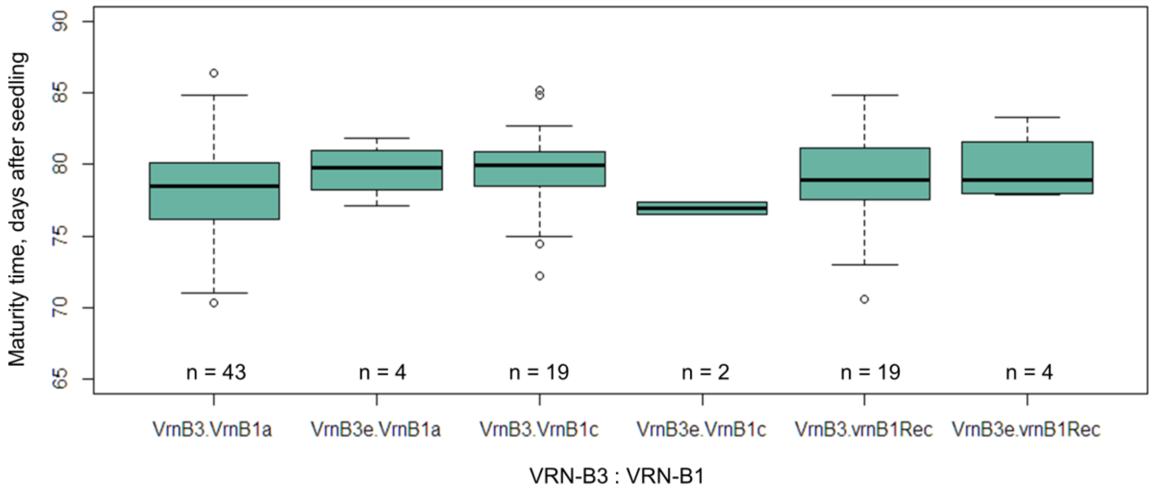

Fig. S2 Boxplots of the maturity time variation for genotypes showing different allele combinations of *Vrn-B1* and *Vrn-B3*. “n” denotes the number of samples in each group.

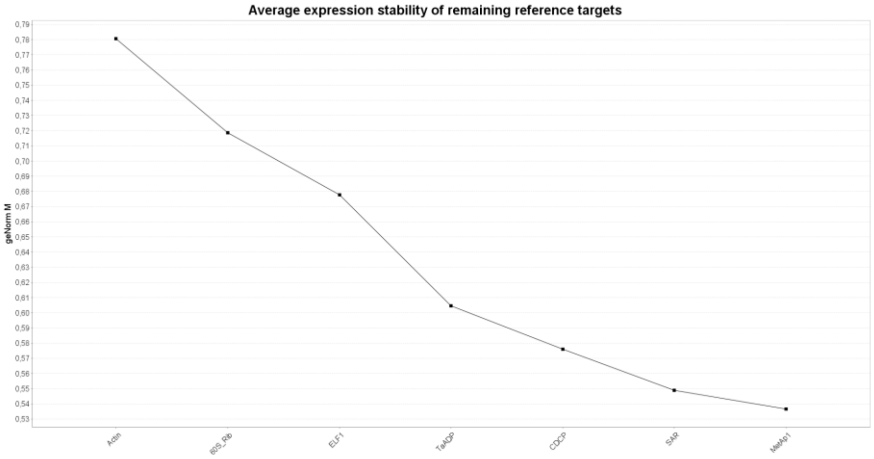

Fig. S3. Expression stability of reference genes, evaluated with geNorm algorithm.

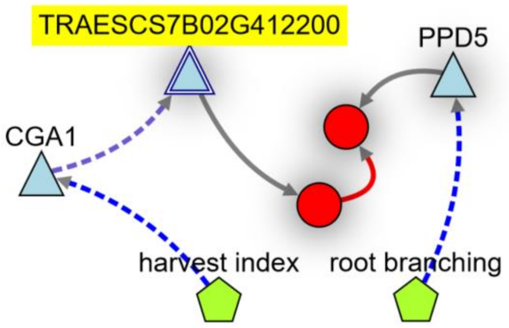

Fig. S4. Gene networks of *TraesCS7B02G412200* (KnetMiner).

Table S5. Annotation of candidate genes based on the functional annotation by IWGSC RefSeq v1.0 and expression with the use of the developmental time course data of the common wheat cultivar Azhurnaya (Ramírez-González et al. 2018).

Gene ID	Peak of the expression	Blast-Hit-Accession	Human-Readable-Description	Pfam-IDs-(Description)	Interpro-IDs-(Description)
TraesCS7B02G391800	in the flag leaf at the flowering stage	tr|A0A0K9Q033|A0A0K9Q033_ZOSMR	Basic-leucine zipper (BZIP) transcription factor family	PF00170: bZIP transcription factor	IPR004827: Basic-leucine zipper domain
TraesCS7B02G396600	in flag leaf and flag leaf sheath from heading to the maturity	tr|B9SUC9|B9SUC9_RICCO	Receptor-kinase, putative	PF13855: Leucine rich repeat; PF00560: Leucine Rich Repeat; PF07714: Protein tyrosine kinase	IPR000719: Protein kinase domain; IPR001245: Serine-threonine/tyrosine-protein kinase, catalytic domain; IPR001611: Leucine-rich repeat; IPR008271: Serine/threonine-protein kinase, active site; IPR011009: Protein kinase-like domain; IPR017441: Protein kinase, ATP binding site; IPR032675: Leucine-rich repeat domain, L domain-like
TraesCS7B02G411800	in flag leaf and flag leaf sheath from heading to the maturity	tr|A0A1D6C995|A0A1D6C995_WHEAT	Patatin	PF01734: Patatin-like phospholipase	IPR002641: Patatin-like phospholipase domain; IPR016035: Acyl transferase/acyl hydrolase/lysophospholipase
TraesCS7B02G401600	in flag leaf during the anthesis and then in embryo proper in ripening stage	tr|C1MYP8|C1MYP8_MICPC	Mitochondrial carrier family	PF00153: Mitochondrial carrier protein	IPR002067: Mitochondrial carrier protein; IPR018108: Mitochondrial substrate/solute carrier; IPR023395: Mitochondrial carrier domain
TraesCS7B02G400600	in flag leaf from heading to the maturity	tr|C7E143|C7E143_ALLCE	CONSTANS-like protein	PF00643: B-box zinc finger; PF06203: CCT motif	IPR000315: B-box-type zinc finger; IPR010402: CCT domain; IPR011011: Zinc finger, FYVE/PHD-type
TraesCS7B02G412200	in flag leaf since the flag leaf stage	tr|A0A0F7GYK8|A0A0F7GYK8_9ASPA	Photosystem II reaction center PsbP family protein	PF01789: PsbP	IPR002683: PsbP family; IPR016123: Mog1/PsbP, alpha/beta/alpha sandwich
TraesCS7B02G399700	in flag leaf since heading to milk grain stage	AT5G38350.1	Disease resistance protein (NBS-LRR class) family	PF00931: NB-ARC domain	IPR002182: NB-ARC; IPR003593: AAA+ ATPase domain; IPR011991: Winged helix-turn-helix DNA-binding domain; IPR027417: P-loop containing nucleoside triphosphate hydrolase; IPR032675: Leucine-rich repeat domain, L domain-like
TraesCS7B02G404300	in flag leaf since full boot to milk grain stage	tr|A0A164TMY6|A0A164TMY6_DAUCA	UDP-glycosyltransferase	PF00201: UDP-glucoronosyl and UDP-glucosyl transferase	IPR002213: UDP-glucuronosyl/UDP-glucosyltransferase
TraesCS7B02G395600	in flag leaf in full boot stage and then in milk grain stage	tr|S4YYQ6|S4YYQ6_HORVV	NAD(P)H-quinone oxidoreductase subunit I, chloroplastic	PF00037: 4Fe-4S binding domain	IPR017896: 4Fe-4S ferredoxin-type, iron-sulphur binding domain; IPR017900: 4Fe-4S ferredoxin, iron-sulphur binding, conserved site
TraesCS7B02G400500	in flag leaf in milk grain ripening and heading stage	tr|A0A1D6D8L7|A0A1D6D8L7_WHEAT	Protein DETOXIFICATION	PF01554: MatE	IPR002528: Multi antimicrobial extrusion protein
TraesCS7B02G398700	in flag leaf in ripening	AT5G02220.1	cyclin-dependent kinase inhibitor		
TraesCS7B02G413400	in flag leaf in heading stage	tr|A0A0K9P8I3|A0A0K9P8I3_ZOSMR	Chaperone protein dnaJ	PF00226: DnaJ domain	IPR001623: DnaJ domain
